# Typhoid Fever

**Published:** 1885-07

**Authors:** T. J. Bennett

**Affiliations:** Austin, Texas


### TYPHOID FEVER.

A CASE WITH UNUSUAL COMPLICATIONS.

*T. J. Bennett, M. D., Austin, Texas.*

Read before the Travis County Medical and Surgical Society.—For Daniel's Texas Medical Journal.

Marie F., aged five years, was attacked with fever on May 16th. The tongue was only slightly coated, the skin rather dry, and the bowels more or less tender and discharging their watery stools several times daily. On the 19th, three days from beginning of attack, the temperature had slowly reached 103o with above symptoms all aggravated. The patient was quiet, but noticing all surrounding objects, and at times was inclined to play, and never refused to take nourishment whenever offered it. Typhoid fever was here diagnosed, which run a uniform course for nine days more, when a severe pharyngitis set in as a complication. Up to this time, the 14th day of the disease, the case had been gradually progressive, but mild. Anorexia, thirst and dryness of fauces and tongue had not reached a very annoying degreee. The bowels were tympanitic most of the time, but evacuations, though frequent, were not offensive. The temperature had not varied over half a degree up to the time of this complication, which immediately increased all the former symptoms, elevating the temperature to above 103o, increasing the dryness of the skin, tongue and faucial surfaces, and producing extreme thirst.and great heat of the mouth and throat.

It is interesting to note with what avidity the patient would crush the small pellets of ice that were placed in her mouth at short intervals.

The tonsils were slightly enlarged, and presented in common with the somewhat thickened mucous surfaces, a darkened color, over all of which was soon spread an abundant, viscid secretion. So thick and gummy was this coating, that it required considerable effort with the finger, wrapped with pieces of soft cloth, to remove it, and so rapidly did it re-accumulate, that a repetition of the cleansing process was necessary every three or four hours.

Deglutition for a time was almost lost; due, perhaps, not altogether to pain at the attempt to swallow, but to the diminished contractive power of the sets of submucous muscles, necessary in the act, from being infiltrated with serum.

The evacuations from the bowels had now become exceedingly offensive and dark, the odor resembling that which comes from gangrenous bowels.

A solution of Argent. Nit. (20 gr. to oz.) was immediately applied to the throat and inflamed surfaces, followed by frequent ablutions of a mixture of sodium hyposulphite, glycerine and acid corbolic. A little Dover's powders occasionally, and the alternate use of counter irritants and hot fomentations about the throat, together with Liq. Am. Acet., to which was added a little Aconite Tr., and an abundance of cold water over the body, to subdue the heat, constituted the principle treatment.

This condition, bad indeed as it was, lasted five days, when, rather suddenly, and to the great pleasure of the physician and friends, all symptoms became ameliorated, and hope was at once entertained of the little sufferer's recovery. But next morning, after observing this apparently favorable change in the case, the patient was found with head thrown back, and spine curved to opisthotonos. She would frequently cry out, and was exceedingly restless. Pot. brom. chloral hyd. and opium, failed to control the pain, tetanic muscular contractions, and rigidity observed in the case. Physostigma was administered, in combination with some of above agents, in full doses, as well as, was used some inter-scapular vesication, and ice to the spine.

With it all, the good constitution and nursing, the patient, in two or three days, began to straighten her neck and spinal colum, and to evince other unmistakable signs of improvement. At no time did the intellect seem impaired; yet there were occasional delirium and incoherent mutterings, but of short duration.

Hope of recovery was again entertained, when still another complication suddenly arose;—that of considerable swelling of the submaxillary glands, which was attended with great pain and heat, which continued two days. This, however, in my experience, is regarded as a favorable crisis in this form of fever.

It will be understood that the nourishment and stimulation of the patient with such food as milk, broths, beef tea, egg-nogg, etc., were well and judiciously attended to, throughout the whole course of the fever, which from beginning to convalescence, was 28 days.

The fact that such complications in typhoid fever are exceedingly rare, and that such results, when complications so formidable do arise, are still more seldom obtained, coupled with the fact that it is a case in practice from which valuable deductions may be drawn by the busy practitioner, constitute my sole apology for reporting it.

